# Supplementary material for: A Global Screen for Assembly State Changes of the Mitotic Proteome by SEC-SWATH-MS
Source: Cell Syst. 2020 Feb 26;10(2):133–155.e6. doi: 10.1016/j.cels.2020.01.001 (PMC7042714; doi:10.1016/j.cels.2020.01.001)

P07900 | HS90A_HUMAN | HSP90AA1 HSP90A HSPC1 HSPCA
Monomer MW [kDa]: 84.66 Monomer expected elution fraction: 42

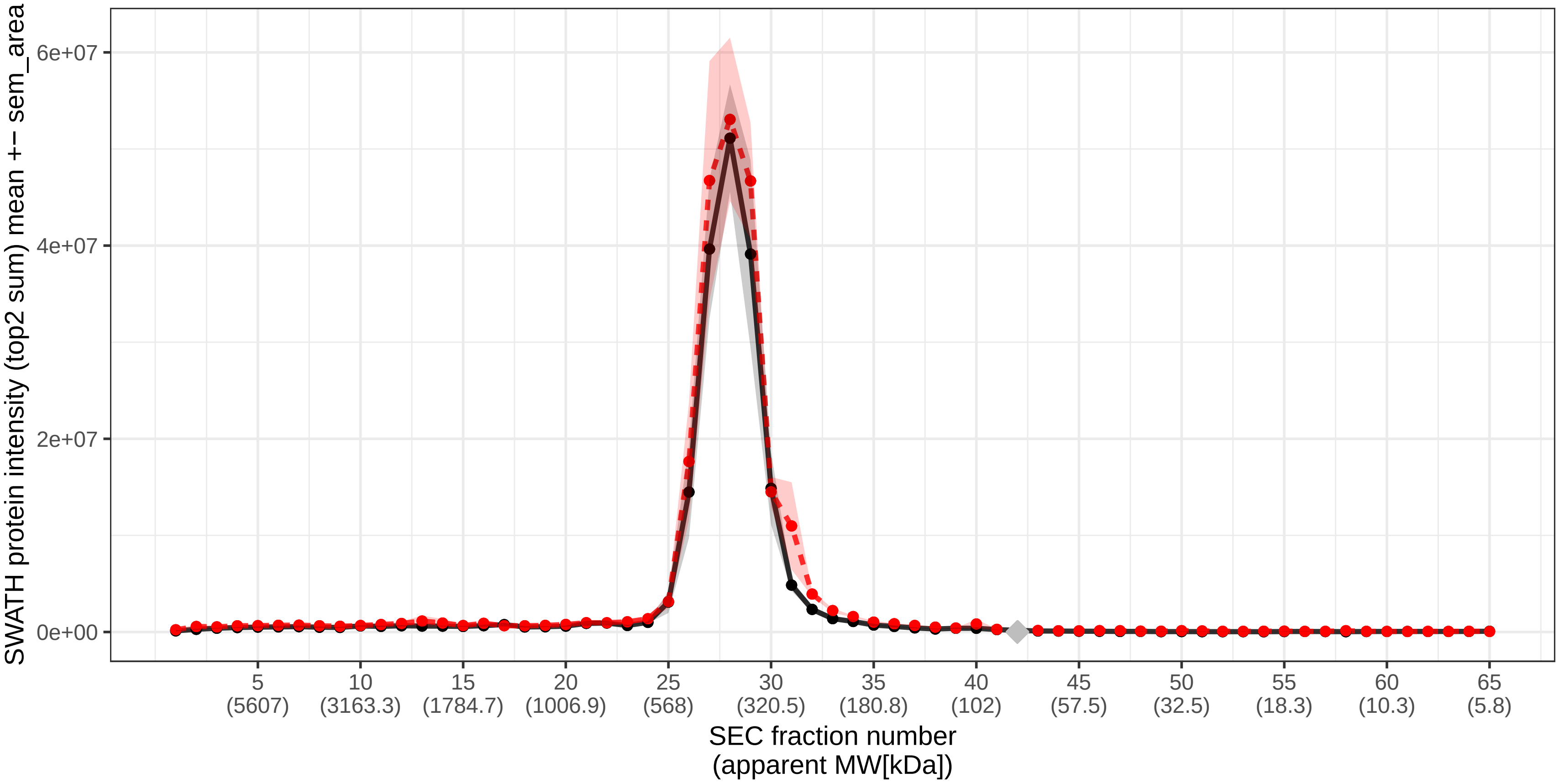

Supplement: Data S1. SEC-SWATH-MS Protein Chromatograms, Related to Figure 1 [file mmc6.zip › SECchrom_P07900_HS90A_HUMAN_HSP90AA1_HSP90A_HSPC1_HSPCA.pdf]
